# Assessment of CT to CBCT contour mapping for radiomic feature analysis in prostate cancer

**DOI:** 10.1038/s41598-021-02154-w

**Published:** 2021-11-23

**Authors:** Ryder M. Schmidt, Rodrigo Delgadillo, John C. Ford, Kyle R. Padgett, Matthew Studenski, Matthew C. Abramowitz, Benjamin Spieler, Yihang Xu, Fei Yang, Nesrin Dogan

**Affiliations:** 1 Department of Radiation Oncology, University of Miami Miller School of Medicine, 1475 NW 12th Ave, Miami, FL 33136 USA; 2 Department of Biomedical Engineering, University of Miami, Coral Gables, FL USA

**Keywords:** Cancer, Anatomy, Biomarkers, Medical research, Oncology, Mathematics and computing

## Abstract

This study provides a quantitative assessment of the accuracy of a commercially available deformable image registration (DIR) algorithm to automatically generate prostate contours and additionally investigates the robustness of radiomic features to differing contours. Twenty-eight prostate cancer patients enrolled on an institutional review board (IRB) approved protocol were selected. Planning CTs (pCTs) were deformably registered to daily cone-beam CTs (CBCTs) to generate prostate contours (auto contours). The prostate contours were also manually drawn by a physician. Quantitative assessment of deformed versus manually drawn prostate contours on daily CBCT images was performed using Dice similarity coefficient (DSC), mean distance-to-agreement (MDA), difference in center-of-mass position (ΔCM) and difference in volume (ΔVol). Radiomic features from 6 classes were extracted from each contour. Lin’s concordance correlation coefficient (CCC) and mean absolute percent difference in radiomic feature-derived data (mean |%Δ|RF) between auto and manual contours were calculated. The mean (± SD) DSC, MDA, ΔCM and ΔVol between the auto and manual prostate contours were 0.90 ± 0.04, 1.81 ± 0.47 mm, 2.17 ± 1.26 mm and 5.1 ± 4.1% respectively. Of the 1,010 fractions under consideration, 94.8% of DIRs were within TG-132 recommended tolerance. 30 radiomic features had a CCC > 0.90 and 21 had a mean |%∆|RF < 5%. Auto-propagation of prostate contours resulted in nearly 95% of DIRs within tolerance recommendations of TG-132, leading to the majority of features being regarded as acceptably robust. The use of auto contours for radiomic feature analysis is promising but must be done with caution.

## Introduction

Radiomics is a promising tool with potential diagnostic, prognostic and predictive powers. The extraction and analysis of quantitative radiological features provides valuable information before, during and after radiation therapy (RT)^1^. Previous studies have linked several radiomic features directly to patient survival^2^. Research has shown the power of radiomics for many disease sites; however, these studies also show variability with respect to imaging modality, reconstruction algorithms, feature selection, and volume of interest (VOI)^3–9^. Several groups have studied the robustness of radiomic features with respect to contouring variability^3–5,10–12^. Contours are typically created by a trained radiation oncologist; however, inter-, and intra-observer contouring variation can still be significant when considering radiomics^10,13^.

A recent study by Yang et al., investigated the impact of contouring variability on PET-based radiomic features for lung cancer^14^. The study demonstrated that the impact of contour uncertainty on PET-based radiomic features varied widely and cautioned predictive use in the context of contouring uncertainty for models involving PET-based radiomic features. A study by Pavic et al., examined intra-observer variation effects on radiomic features extracted from CT images^12^. This study extracted a total of 137 radiomic features from planning CT images of head and neck cancer patients and warned that variation in delineation can significantly affect some radiomic features.

On-board imaging (OBI) utilizing megavoltage (MV) and kilovoltage (kV) cone beam CT (CBCT) is a widely used imaging technique for daily patient bony alignment and prostate marker alignment^15^. The prostate can deform and rotate daily due to differential bladder and rectal filling resulting in suboptimal dosimetry over the course of treatment^16^. By utilizing CBCT setup images, changes in anatomy can be accounted for at the time of treatment delivery^17^. Deformable image registration (DIR) can automatically propagate the contours drawn on the planning CT (pCT) to daily CBCT images, accounting for the anatomical changes and allowing adaptive radiotherapy (ART)^18,19^. The American Association of Physicists in Medicine (AAPM) Task Group 132 (TG-132) provides recommendations on the use of image registration and fusion algorithms and provides quantitative methods of evaluating DIR accuracy^20^.

Various studies have investigated the accuracy of the DIR algorithms for the automatic creation of contours (auto contours) for prostate cancer; however, these studies often used very small sample sizes^18,21–23^. A study by Woerner et al.^18^, evaluated the DIR performance from pCT to CBCT that was acquired near the end of treatment for 6 prostate, 5 head and neck and 5 pancreas patients. The small sample size of their study limited their analysis to organs at risk (OAR). The authors cautioned the use of automatic DIR workflows to perform contour deformation to assess the changes during the course of treatment. Another study by Thor et al., evaluated DIR performance from pCT to CBCT through the course of prostate cancer treatment for 5 patients^24^. The study concluded that more advanced imaging and/or DIR algorithms should be developed to confidently use DIR workflows for contour deformation.

Unlike CT, PET and MR images that are used for diagnostic and treatment planning purposes, CBCT images are used for daily patient setup prior to radiation delivery and are collected as the current standard of care on a daily basis. Most radiomic studies performed thus far have been evaluated in a pre-treatment setting and are lacking a knowledge of early tumor response to therapy, hindering the possibility for timely treatment adaptation. Hence, the day-to-day radiomic feature changes of the tumor obtained from CBCT may offer a possibility of treatment adaptation during the early course of treatment, distinct from radiomic predictions derived in the pre-treatment settings. These features can be examined for their use in early response assessment.

The automatic propagation of pCT contours to daily CBCT can also be used in the context of radiomics. However, to our knowledge, the robustness of radiomic features to varying prostate contours on daily CBCT’s has not been previously examined. By updating radiomic feature derived data on a more frequent basis, as could be done through utilizing daily CBCT derived data, radiomic features can help influence clinical decision making. The goal of this study is to utilize a commercially available DIR algorithm to deform manual prostate contours to the daily CBCTs and determine the robustness of radiomic features to DIR-based contour propagation.

## Methods and materials

### Patient selection

Twenty-nine prostate cancer patients who were treated with volumetric modulated arc therapy (VMAT) and had daily CBCT images were considered. The ethical approval for this study was obtained from the University of Miami Institutional Review Board (IRB). Written informed consent was obtained from all patients in this study. The data was retrospectively collected and analyzed. All methods undertaken in this work were carried out in accordance with the relevant guidelines and regulations. One patient was excluded due to prosthetic implants causing extremely poor image quality. Each patient was treated using conventional fractionation, consisting of 28–40 fractions, totaling 1,010 total fractions. Prostate volumes ranged from 15 cm^3^ to 92 cm^3^.

### Image acquisition and manual contouring

Planning CT (pCT) images were acquired using Somatom Definition AS and Sensation Open (Siemens Healthineers AG, Germany), and/or Gemini TF TOF 64 (Philips, Netherlands) for each patient. The mean field-of-view (FOV) was 670 mm with a range of 492–800 mm, reconstructed with dimensions of 512 × 512 pixels, a thickness of 2 mm, and an average in-slice pixel size of 1.3 mm with a range of 0.9–1.6 mm. On the day of treatment, each patient was imaged with the same FOV using kV CBCT with 465 slices, voxel sizes of (0.9 mm, 0.9 mm, 2.0 mm) and reconstructed with dimensions of (512, 512, 232). Each patient was imaged in supine position. All 28 patients had 4 gold fiducial markers implanted in the prostate prior to imaging. Prostate volumes were manually contoured on pCT and on daily CBCT setup images by the same expert radiation oncologist who has 4 years of experience in contouring prostate cases to eliminate interobserver variation. The pCT images and daily CBCT setup images, including manually drawn contours, were uploaded to a commercial image registration software (Velocity Advanced Imaging, ver. 4.1, Varian Medical Systems, Palo Alto, CA).

### Deformable image registration and delineation propagation

**Figure 1:**
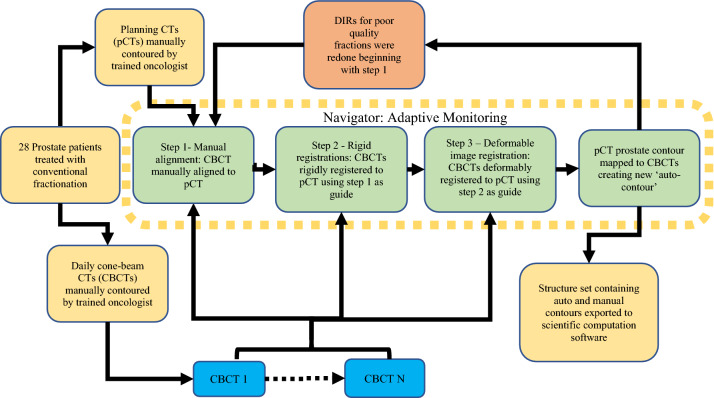
Deformable image registration and delineation propagation workflow. All steps included in the Adaptive Monitoring Navigator on Velocity are inside the dotted-line box. The re-evaluation of poor-quality fractions (DSC < 0.75, MDA > 3.5 mm) is shown in the light-red box. Exportation of data to MATLAB for data extraction and analysis is done in the final green box.

The DIR of pCT to daily CBCT images were performed using an Adaptive Monitoring tool available in the commercial image registration software. Figure 1 shows the DIR and delineation propagation workflow. DIR creation utilized the ‘Adaptive Monitoring’ navigator, which is comprised of three steps: (1) manual alignment, (2) rigid registration, (3) deformable registration. During the manual alignment step, a manual rigid alignment between the CBCT image and the pCT image was made using bony anatomy and the implanted gold fiducials as a guide. These were done independently of clinical set-up shifts for consistency. In step 2 the region of interest was adjusted to include the prostate and, using the manual alignment created in step 1 as a starting point, a rigid registration was created by the software. Step 3 uses the rigid registrations of step 2 as a reference to create DIR’s. After the rigid registration, the DIR algorithm was utilized to deformably register the pCT to the daily CBCTs. The DIR algorithm uses an intensity-based B-spline algorithm based on the Mattes formulation; the details have been described elsewhere^14,25^. For poor quality deformations, the deformable image registration workflow was repeated with smaller ROIs (67 fractions). Poor quality deformations were defined as a DSC < 0.75 and MDA > 3.5 mm, just beyond the TG-132 tolerance recommendations. For the three fractions that continued to produce poor quality deformations, a structure-guided deformation (hybrid DIR) was employed.

### Assessment of deformable image registration

The DIRs from pCT to daily CBCTs were done using both qualitative and quantitative assessment metrics. First, a visual assessment of deformation vector fields (DVFs) was carried out to ensure that the DIR transformation was physically and anatomically reasonable. The locations of anatomical landmarks (e.g., bones) and fiducial markers were visually inspected on fused images of the pCT and CBCT to verify that they matched. DIRs were refined by adjusting the ROI around the prostate to improve DIR accuracy^26^, or by applying a structure-guided deformation.

The quantitative assessment of the registered prostate contours was done using several metrics. Dice similarity coefficient (DSC) is a statistical measure of contour overlap with 0 being no overlap and 1 being a perfect match^27^. Distance to agreement (DTA) between two contours, sometimes referred to as distance to conformity, is the shortest distance from a given point on the surface of one contour to the surface of the other contour. Mean distance to agreement (MDA) is the mean of all DTA distances^28^. The geometric centers of both the auto and manual contours on the CBCTs were calculated and used to determine the difference in center of mass position (Δ*CM*). The difference in volume between the auto and manual contour (∆Vol) was also evaluated for all 1,010 fractions. For a smaller sub-sample of 10 patients, on a bi-weekly basis (totaling 47 fractions), the Jacobian determinant (JD) was computed. Jacobian determinant values corresponding to volume expansion, no volume change and volume reduction are > 1, 1, and < 1, respectively. JD values equal to or less than zero correspond to non-physical transformations which are indications of a poor DIR^20^. DSC, MDA, Δ*CM*, and ∆Vol were calculated between the auto and manual prostate contours for all 1,010 fractions. TG-132 defines a clear method for assessing DIR algorithms^20^. In this work, the tolerances defined in the TG-132 were used for quantitative assessment of the DIRs.

### Radiomic features

To study the impact of contouring variability due to the DIR on CBCT radiomic features in the prostate a total of 46 radiomic features derived from 6 different classes were analyzed for all 1,010 fractions. Additionally, a subpopulation consisting of 149 fractions was identified having ∆Vol > |10%| to study the impact of larger contour variability on CBCT radiomic features. Radiomic features were extracted using the procedure described in Delgadillo et al.^11^, including Gray-Level Co-occurrence Matrices (GLCM), Neighborhood Gray-Tone Difference Matrix (NGTDM), Gray-Level Run-Length Matrices (GLRLM), Gray-Level Size Zone Matrices (GLSZM), Morphological and statistical features^29–31^. Each radiomic feature is distinguished by the image biomarker standardization initiative (IBSI) code^32^.

Percent difference in radiomic feature derived data (%∆RF) was compared to DSC to assess radiomic feature dependency on contouring variability using Spearman’s rank correlation coefficient (*ρ*)^33,34^. These correlations were classified as weak if |*ρ*|< 0.4, moderate if 0.4 ≤|*ρ*|< 0.6, relatively strong if 0.6 ≤|*ρ*|< 0.8, and strong if 0.8 ≤|*ρ*|^33^.

Lin’s concordance correlation coefficient (CCC) was computed for all 46 radiomic features between the auto and manual contours to find the strength of correlation^35^, with a perfectly linear relationship equal to 1 and no relation being 0^34,36^. Adapting the classification scheme proposed by McGraw et al.^37^, radiomic features were classified as robust with CCC > 0.90, acceptable with 0.75 < CCC < 0.90, and uncertain with CCC < 0.75.

Additionally, mean absolute percent difference in radiomic feature derived data (mean |%∆|RF) was used to evaluate the stability of radiomic features to differences in prostate contours. Radiomic features were independently classified as robust with mean |%∆|RF < 5%, acceptable with 5% < mean |%∆|RF < 15%, and uncertain with 15% < mean |%∆|RF < 50%. Processing and analysis were performed using scientific computation software (MATLAB, ver. 2018b, Math-Works Inc., Natick, MA).

## Results

### Assessment of prostate contour accuracy

**Figure 2:**
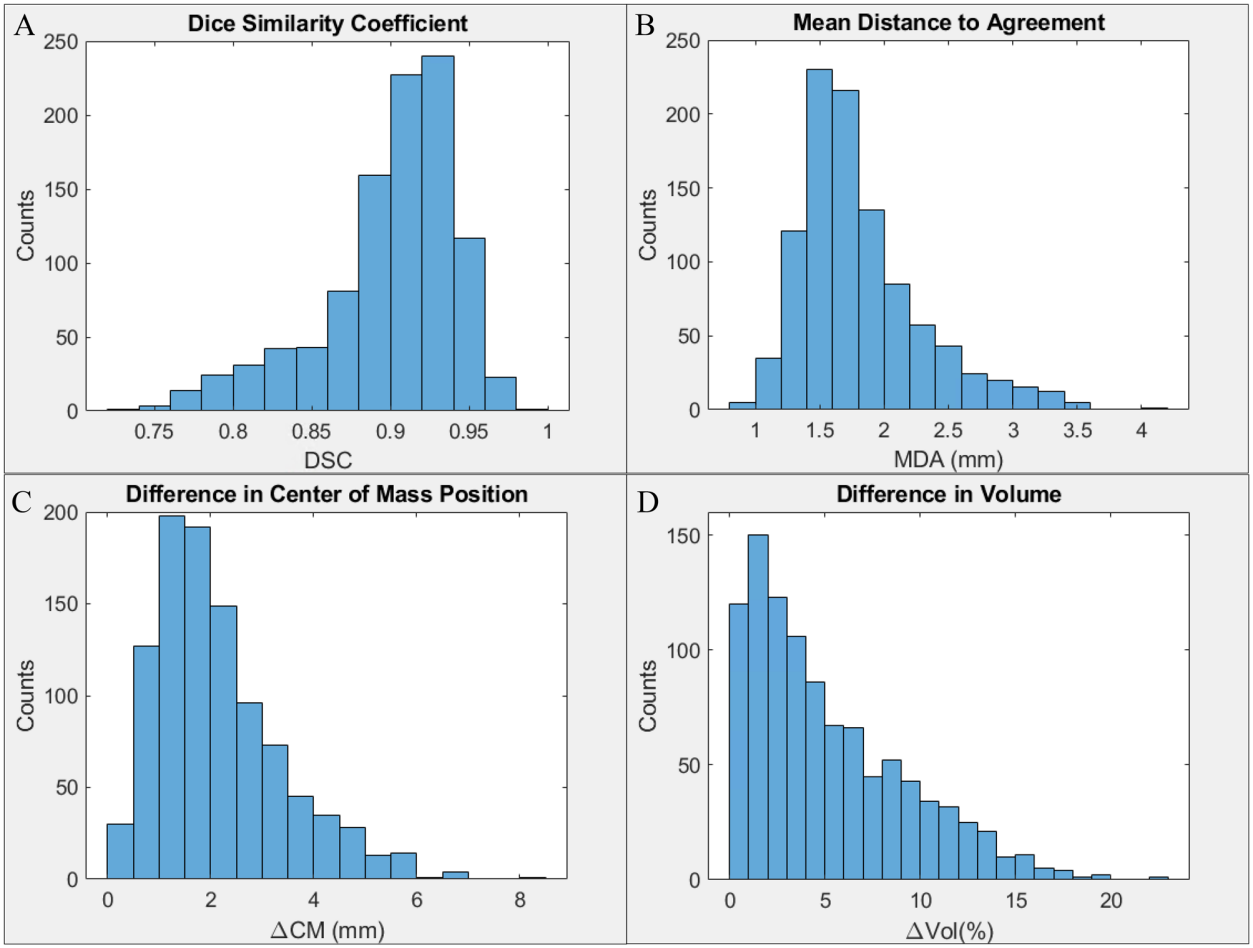
Histograms of (**A**) DSC, (**B**) MDA, (**C**) ΔCM and (**D**) %Δ volume between the auto and manual contours for all 1010 fractions are shown in (**A**)–(**D**) respectively.

Table 1 summarizes the mean, standard deviation and range for DSC, MDA, Δ*CM*, ∆Vol, JD minimum and JD maximum between auto and manual prostate contours, and Fig. 2 shows the distribution of DSC, MDA, ΔCM and ΔVol over 1010 fractions. The mean DSC of all 1,010 fractions, 0.90 ± 0.04, is within the TG-132 recommended DSC value of ~ 0.8 to 0.9. A total of 42 fractions had DSC < 0.8, below the lower tolerance recommendation of TG-132.

**Table 1:** Similarity metrics between auto and manual contours.

	Mean	SD	Range	TG-132 recommendation
Dice similarity coefficient	0.90	0.04	(0.74, 0.98)	∼ 0.8 to 0.9
Mean distance to agreement (mm)	1.80	0.50	(0.88, 4.16)	∼ 2 to 3
Difference in center of mass position (mm)	2.17	1.38	(0.06, 8.18)	NA
Difference in volume (%)	5.10	4.10	(0.06, 22.8)	NA
Jacobian minimum	0.77	0.18	(0.25, 0.97)	> 0
Jacobian maximum	1.30	0.23	(1.02, 1.97)	NA

The mean MDA of 1.81 ± 0.47 mm is well within the TG-132 recommended ~ 2 to 3 mm. A total of 33 fractions had MDA > 3 mm with a maximum MDA of 4 mm. The mean Δ*CM* found to be 2.17 ± 1.26 mm and mean ∆Vol of 5.1 ± 4.1%. The mean minimum and mean maximum values of the JD were found to be 0.77 ± 0.18 and 1.31 ± 0.23 respectively, with no JD values ≤ 0 (Table 1).

### Impact of contouring variability on radiomic features

**Figure 3:**
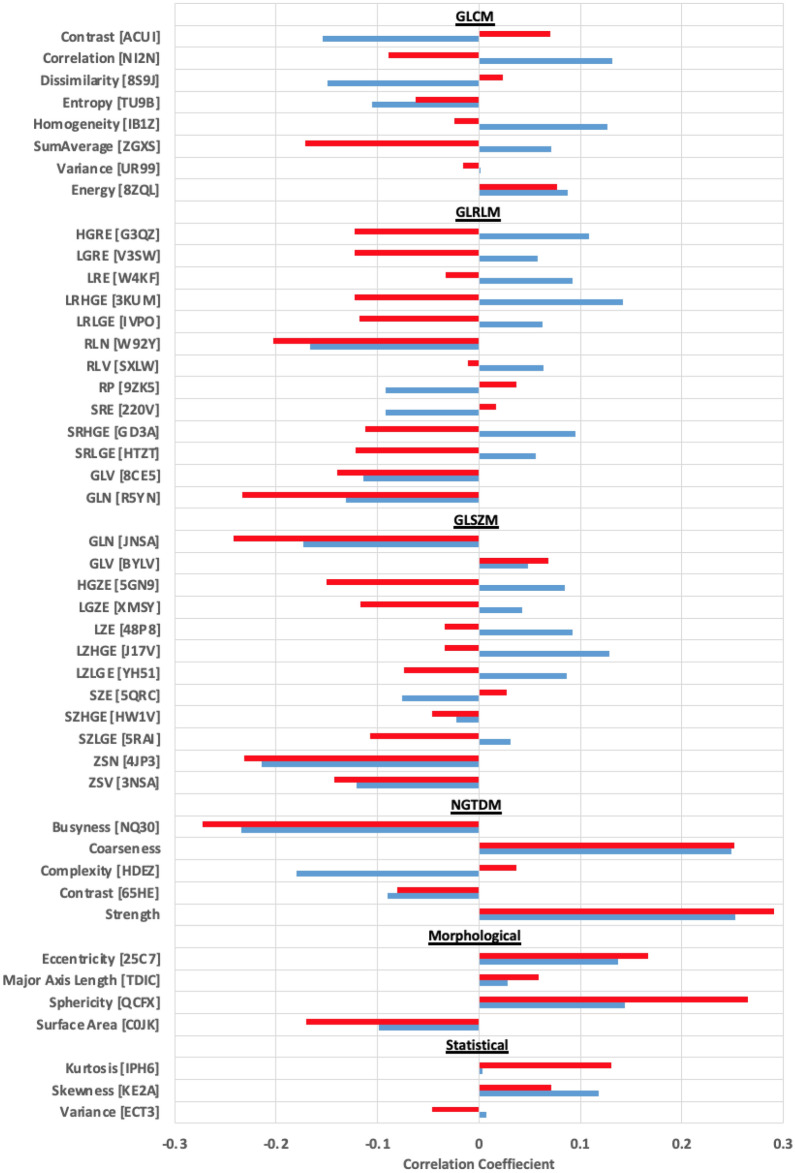
Spearman’s rank correlation coefficient between the mean absolute percent difference in radiomic feature derived data (%∆RF) between auto and manual contours plotted against Dice similarity coefficient (DSC), stratified by class. This was done for two populations, all fractions (blue) and for fractions with ΔVol > |10%| (red).

Spearman rank correlation coefficient for %∆RF versus DSC for each radiomic feature is shown in Fig. 3. As previously mentioned, a subpopulation of fractions with ∆Vol > |10%| was also considered, results from these fractions are plotted in red (Fig. 3). All 46 radiomic features were classified as having a weak correlation between %∆RF and DSC with |*ρ*|< 0.4 for both populations under consideration (all fractions and sub-population of fractions with ∆Vol >|10%|).

Table 2 displays Lin’s concordance correlation coefficient (CCC) for all 46 radiomic features. Using the classification scheme mentioned earlier, 30 of 46 radiomic features were classified as robust, 8 radiomic features were classified as acceptable, and 8 radiomic features were classified as uncertain (Table 2). Neighborhood Gray-Tone Difference Matrix (NGTDM) and Gray-Level Co-occurrence Matrices (GLCM) had the highest mean CCC with values of 0.963 and 0.943, respectively.

**Table 2:**
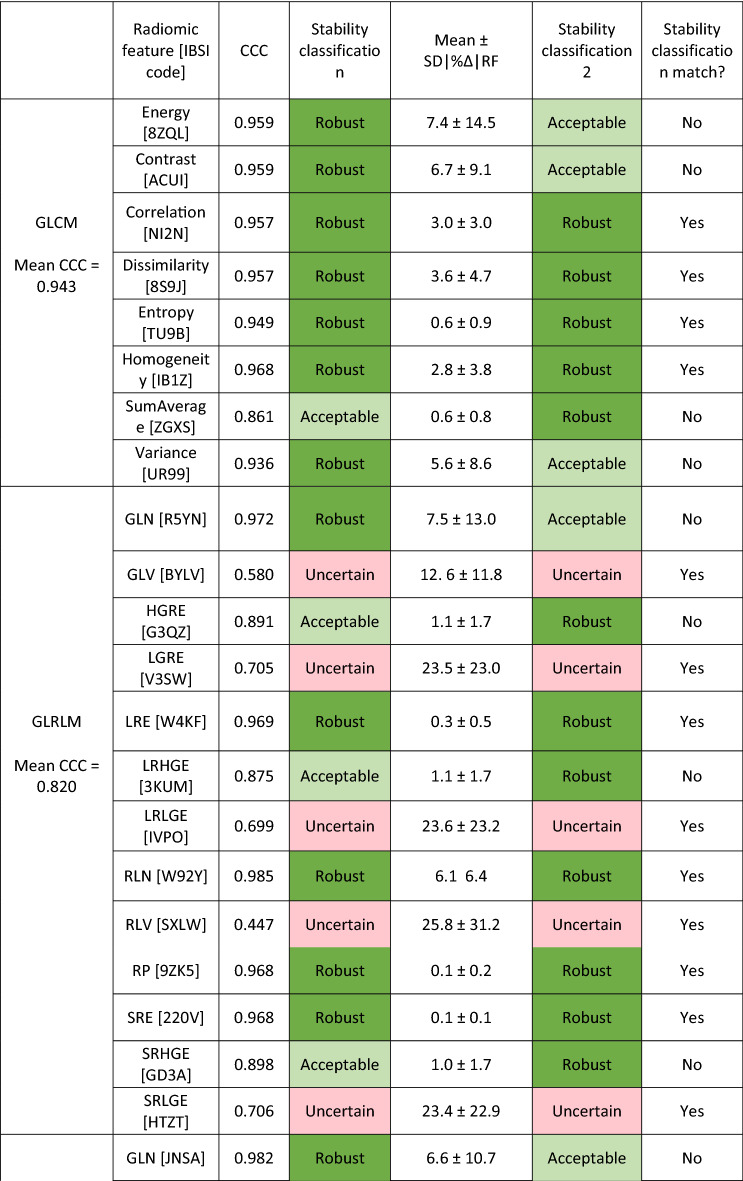

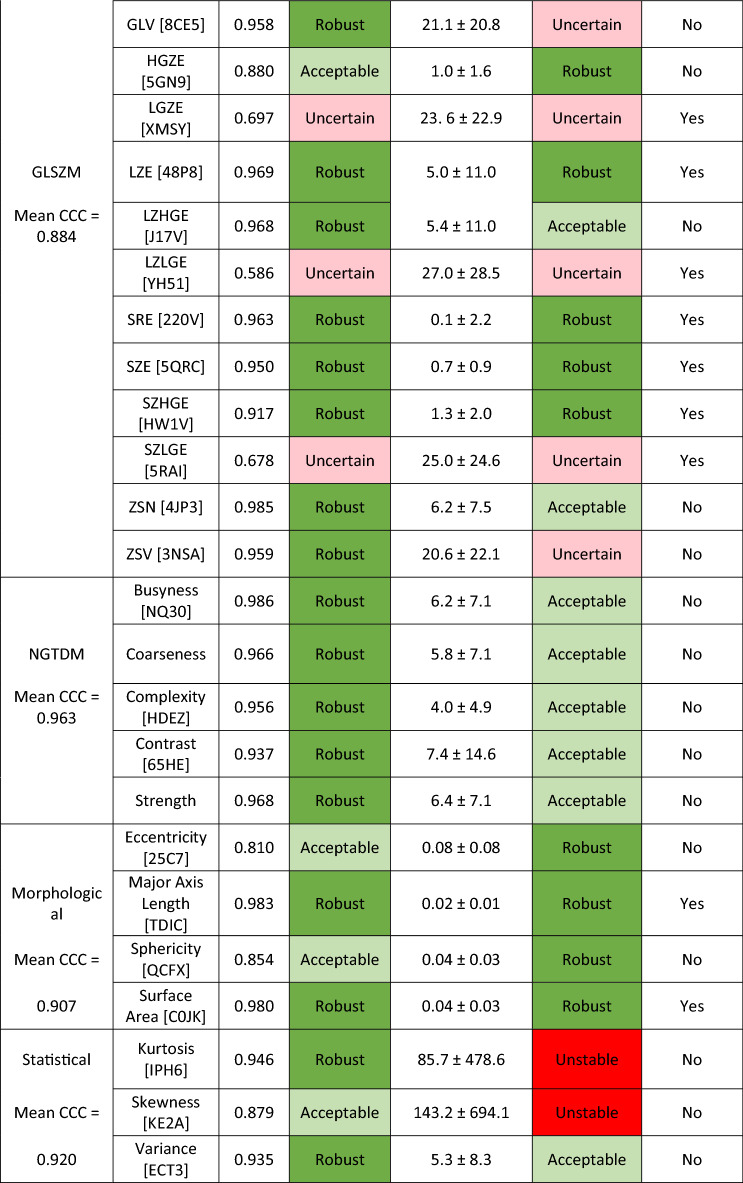
Lin’s concordance correlation coefficient (CCC) and mean absolute percent difference in radiomic feature derived data (|%∆|RF) between auto and manual contours for all radiomic features.

Corresponding stability classifications were given according to each independently and compared for consistency. CCC values classified as robust CCC > 0.90, acceptable with 0.75 < CCC < 0.90, and uncertain with CCC < 0.75. Radiomic features were classified according to mean absolute percent difference in radiomic feature derived data (|%∆|RF) as robust with mean |%∆|RF < 5%, acceptable with 5% < mean |%∆|RF < 15%, and uncertain with 15% < mean |%∆|RF < 50%.

Independent classification according to mean |%∆|RF can also be seen in Table 2 separated by class. In total 21 of 46 radiomic features were classified as robust and 7 of the 13 GLRLM radiomic features were classified as robust. 14 radiomic features were classified as robust according to both CCC and mean |%∆|RF. 24 radiomic features did not have matching stability classifications when comparing the classifications from CCC and mean |%∆|RF.

## Discussion

### DIR and contour accuracy

**Figure 4:**
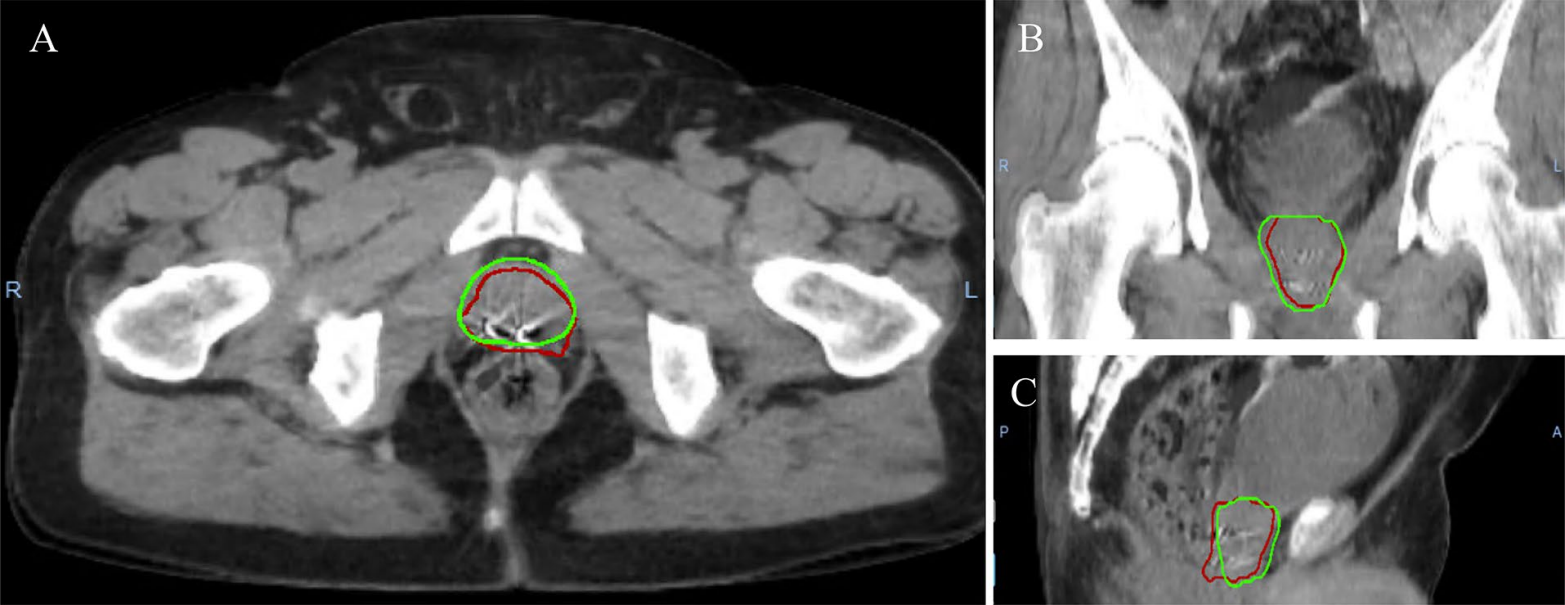
Automatically generated prostate contour (red) and manually drawn contour (green) for sample patient with poor match statistics (DSC<0.8 and MDA>3). Shown axial slice (**A**), coronal slice (**B**), and sagittal slice (**C**).

Mean DSC and mean MDA of the current study (Table 1) are both well within the TG-132 recommended tolerances, indicating that the DIR workflow followed here can produce accurate prostate contours. A study done by Forde et al., found that 37% of contours created through DIR had a DSC < 0.7^5^. The DIR algorithm of our study, having no contours with DSC < 0.7, outperformed the one used by Forde et al. which used an earlier version of Varian’s auto contouring (SmartSegmentation version 15.5) which used an atlas-based algorithm. However, 4.1% of contours created in this study still had DSC below the TG-132 recommendation of 0.8—further inspection of these fractions found acceptable displacement vector fields and acceptable contours as shown in Fig. 4 for a poor performing fraction (DSC = 0.79, MDA = 3.02).

While *ΔCM* and ∆Vol are not included in the TG-132 recommendation, both are useful indications of auto and manual contour matching. A previous study by Studenski et al., considered 16 patients on hypo-fractionated schemes and found percent difference in prostate volume from contours created through a DIR workflow to be < 10%^38^, and a previous study by Forde et al., which looked at inter-observer delineation variability and its impacts on radiomic feature robustness found the interobserver variation in contour sizes were as high as 80%^5^. In the study presented here, 149 of the 1,010 fractions had a difference in volume between the auto and manual contours (∆Vol) > 10% (Table 1). Large discrepancies in volume indicate a poor DIR, however, the mean DSC of these 149 fractions was 0.88, and visual inspections resulted in acceptable contours.

The radiomic features belonging to the morphological features class can also be used as a measure of contour accuracy. As can be seen in Table 2, the mean |%∆|RF for all 4 morphological features is less than 0.1%. These low mean absolute percent difference in radiomic feature derived data for the morphological feature class is another indication that the automatically generated contour and the manual contour are in good agreement.

### Radiomic feature robustness

Spearman rank correlation coefficient for %∆RF versus DSC for each radiomic feature as shown in Fig. 3, shows that all 46 radiomic features were classified as having a week correlation (for both populations under consideration). A low Spearman rank correlation coefficient between %∆RF versus DSC simply highlights the complex interplay between radiomic feature derived data of differing contours. That is, a large difference in contour does not necessarily translate to a large difference in radiomic feature derived data, and a small difference in contour does not necessarily translate to a small difference in radiomic feature derived data.

Higher Lin’s concordance correlation coefficient (CCC) translates to robust radiomic features. As seen from Table 2, the NGTDM class radiomic features had the highest mean CCC of 0.963, while GLRLM class radiomic features had the lowest mean of 0.820. Based on this, NGTDM was the most robust class of radiomic features, while GLRLM was the least robust class of radiomic features when considering differences in prostate contours. In contrast to our study, Rizzetto et al., found that GLRLM was the most robust to differing contours, considering colorectal liver metastases contours^10^. These incongruous results may be due to the differing locations within the body (prostate versus liver) and/or the differing contour sizes, but only further the idea that the robustness of radiomic features should be evaluated as they can vary by location. Future radiomic studies should consider the location specific radiomic feature robustness, as the radiomic feature derived data has varying dependence on contour as seen in this study and others^3–5,10–12^.

Similar to the results of this study, a different study done by Yang et al., evaluating radiomic feature robustness taken from PET images of lung cancer patients, found only weak or moderate correlations between %∆RF and DSC^14^. A weak correlation implies a more complex interplay between the delineation of the volume and the contents of the volume. While two contours of a volume may have a high DSC, the percent difference in radiomic feature-derived data from the two contours may be very different.

Mean |%∆|RF alone was also used to evaluate the stability of radiomic features to differences in prostate contours. Forde et al., found that GLRLM had the smallest mean |%∆|RF considering parotid gland contours^5^. The results of Forde et al. agree with the work presented here, finding 7 of the 13 GLRLM radiomic features were classified as robust, the highest performing class.

Severe discrepancies between CCC and mean |%∆|RF do occur. For example, statistical features of ‘Skewness’ and ‘Kurtosis’ had CCC values of 0.879 and 0.946 respectively and mean ± SD in |%∆|RF of 143.2 ± 694.1 and 85.7 ± 4786, respectively. If the auto contour radiomic feature derived data and manual contour radiomic feature derived data have similar means and standard deviations over all fractions, CCC will be high and indicate high similarity. However, in the same situation, |%∆|RF per auto and manual contour pair can be large and varying. Thus, leading to situations where a radiomic feature has both a desirable high CCC and an undesirable high mean |%∆|RF.

## Conclusions

This study demonstrated that an intensity-based DIR algorithm applied to daily CBCTs is sufficiently robust and accurate to meet the recommendations of TG-132 for prostate cancer. The radiomic features derived from DIR-generated auto contours and manually drawn contours were acceptably similar for 22 of 46 radiomic features. However, there is a varying dependence on contours from one radiomics class to another and from one radiomic feature to another. Weak correlations between mean |%∆|RF and DSC imply a complex interplay of volume and contents when considering radiomic feature data extraction from prostate contours that requires further insight.

## References

[1] Aerts HJWL (2014). Decoding tumour phenotype by noninvasive imaging using a quantitative radiomics approach (vol 5, pg 4006, 2014). Nat. Commun..

[2] Zhang H (2013). Locally advanced squamous cell carcinoma of the head and neck: CT texture and histogram analysis allow independent prediction of overall survival in patients treated with induction chemotherapy. Radiology.

[3] Zhao B (2021). Understanding sources of variation to improve the reproducibility of radiomics. Front. Oncol..

[4] Traverso A (2020). Sensitivity of radiomic features to inter-observer variability and image pre-processing in Apparent Diffusion Coefficient (ADC) maps of cervix cancer patients. Radiother. Oncol..

[5] Forde E (2021). Influence of inter-observer delineation variability on radiomic features of the parotid gland. Phys. Med..

[6] Ford J (2018). Quantitative radiomics: Impact of pulse sequence parameter selection on MRI-based textural features of the brain. Contrast Media Mol. Imaging.

[7] Yang F (2018). Evaluation of radiomic texture feature error due to MRI acquisition and reconstruction: A simulation study utilizing ground truth. Physica Med..

[8] Yang F (2018). Magnetic resonance imaging (MRI)-based radiomics for prostate cancer radiotherapy. Transl. Androl Urol..

[9] Simpson G (2020). Impact of quantization algorithm and number of gray level intensities on variability and repeatability of low field strength magnetic resonance image-based radiomics texture features. Physica Med..

[10] Rizzetto F (2020). Impact of inter-reader contouring variability on textural radiomics of colorectal liver metastases. Eur. Radiol. Exp..

[11] Delgadillo R (2021). Repeatability of CBCT radiomic features and their correlation with CT radiomic features for prostate cancer. Med. Phys..

[12] Pavic M (2018). Influence of inter-observer delineation variability on radiomics stability in different tumor sites. Acta Oncol..

[13] Qiao H (2017). Interobserver variability in tumor contouring affects the use of radiomics to predict mutational status. J. Med. Imaging.

[14] Yang F (2020). Impact of contouring variability on oncological PET radiomics features in the lung. Sci. Rep..

[15] Lawson JD (2008). Early clinical experience with kilovoltage image-guided radiation therapy for interfraction motion management. Med. Dosim..

[16] Xu Y (2021). Assessment of daily dose accumulation for robustly optimized intensity modulated proton therapy treatment of prostate cancer. Physica Med..

[17] Yan D (1997). Adaptive radiation therapy. Phys. Med. Biol..

[18] Woerner AJ (2017). Evaluation of deformable image registration-based contour propagation from planning CT to cone-beam CT. Technol. Cancer Res. Treat..

[19] Simon A (2015). *Roles of deformable image registration in adaptive RT: From contour propagation to dose monitoring.* Annu. Int. Conf. IEEE Eng. Med. Biol. Soc..

[20] Brock KK (2017). Use of image registration and fusion algorithms and techniques in radiotherapy: Report of the AAPM Radiation therapy committee task group no. 132. Med. Phys..

[21] Kim J (2013). A novel approach for establishing benchmark CBCT/CT deformable image registrations in prostate cancer radiotherapy. Phys. Med. Biol..

[22] Motegi K (2019). Usefulness of hybrid deformable image registration algorithms in prostate radiation therapy. J. Appl. Clin. Med. Phys..

[23] Nie K (2016). Performance variations among clinically available deformable image registration tools in adaptive radiotherapy—How should we evaluate and interpret the result?. J. Appl. Clin. Med. Phys..

[24] Thor M (2011). Deformable image registration for contour propagation from CT to cone-beam CT scans in radiotherapy of prostate cancer. Acta Oncol..

[25] Kadoya N (2014). Evaluation of various deformable image registration algorithms for thoracic images. J. Radiat. Res..

[26] Lin H (2010). SU-GG-I-109: A quantitative evaluation of velocity AI deformable image registration. Med. Phys..

[27] Dice LR (1945). Measures of the amount of ecologic association between species. Ecology.

[28] Chalana V, Kim Y (1997). A methodology for evaluation of boundary detection algorithms on medical images. IEEE Trans. Med. Imaging.

[29] Thibault G (2013). Shape and texture indexes application to cell nuclei classification. Int. J. Pattern Recogn. Artif. Intell..

[30] Haralick RM (1979). Statistical and structural approaches to texture. Proc. IEEE.

[31] Stoecker WV, Chiang C-S, Moss RH (1992). Texture in skin images: Comparison of three methods to determine smoothness. Comput. Med. Imaging Gr..

[32] Zwanenburg A (2020). The image biomarker standardization initiative: Standardized quantitative radiomics for high-throughput image-based phenotyping. Radiology.

[33] Kendall MG (1938). A new measure of rank correlation. Biometrika.

[34] Spearman C (1907). Demonstration of formule for true measurement of correlation. Am. J. Psychol..

[35] Liu J (2016). Correlation and agreement: Overview and clarification of competing concepts and measures. Shanghai Arch. Psychiatry.

[36] Lawrence IKL (1989). A concordance correlation coefficient to evaluate reproducibility. Biometrics.

[37] McGraw KO, Wong SP (1996). "Forming inferences about some intraclass correlations coefficients": Correction. Psychol. Methods.

[38] Studenski MT (2020). Margin verification for hypofractionated prostate radiotherapy using a novel dose accumulation workflow and iterative CBCT. Phys. Med..

